# Role of rs873601 Polymorphisms in Prognosis of Lung Cancer Patients Treated with Platinum-Based Chemotherapy

**DOI:** 10.3390/biomedicines11123133

**Published:** 2023-11-24

**Authors:** Ting Zou, Jun-Yan Liu, Qun Qin, Jie Guo, Wen-Zhi Zhou, Xiang-Ping Li, Hong-Hao Zhou, Juan Chen, Zhao-Qian Liu

**Affiliations:** 1 Department of Pharmacy, National Institution of Drug Clinical Trial, Xiangya Hospital, Central South University, Changsha 410008, China; zouting@csu.edu.cn (T.Z.); qinqun8087@hotmail.com (Q.Q.); guojiegina@126.com (J.G.); ellery127@163.com (W.-Z.Z.); xylxping@126.com (X.-P.L.); 2 Department of Clinical Pharmacology, Hunan Key Laboratory of Pharmacogenetics, Xiangya Hospital, Central South University, Changsha 410078, China; hhzhou2003@163.com; 3 National Clinical Research Center for Geriatric Disorders, Xiangya Hospital, Central South University, Changsha 410008, China; 4 International Science and Technology Innovation Cooperation Base for Early Clinical Trials of Biological Agents in Hunan Province, Changsha 410008, China; 5 Department of Orthopaedics, Xiangya Hospital, Central South University, Changsha 410008, China; liujunyana@126.com

**Keywords:** lung cancer, platinum-based chemotherapy, *ERCC5*, genetic polymorphism, prognosis

## Abstract

Background: Lung cancer is still the most lethal malignancy in the world, according to the report of Cancer Statistics in 2021. Platinum-based chemotherapy combined with immunotherapy is the first-line treatment in lung cancer patients. However, the 5-year survival rate is always affected by the adverse reactions and drug resistance caused by platinum-based chemotherapy. DNA damage and repair system is one of the important mechanisms that can affect the response to chemotherapy and clinical outcomes in lung cancer patients. Objective: The objective of this study is to find the relationship between the polymorphisms of DNA repair genes with the prognosis of platinum-based chemotherapy in lung cancer patients. Patients and Methods: We performed genotyping in 17 single nucleotide polymorphisms (SNPs) of Excision Repair Cross-Complementation group (*ERCC*) genes and X-ray Repair Cross-Complementing (*XRCC*) genes of 345 lung cancer patients via Sequenom MassARRAY. We used Cox proportional hazard models, state, and plink to analyze the associations between SNPs and the prognosis of lung cancer patients. Results: We found that the *ERCC5* rs873601 was associated with the overall survival time in lung cancer patients treated with platinum-based chemotherapy (*p* = 0.031). There were some polymorphisms that were related to the prognosis in specific subgroups of lung cancer. Rs873601 showed a great influence on the prognosis of patients more than 55 years, Small Cell Lung Cancer (SCLC), and smoking patients. Rs2444933 was associated with prognosis in age less than 55 years, SCLC, metastasis, and stage III/IV/ED patients. Rs3740051 played an important role in the prognosis of SCLC and metastasis patients. Rs1869641 was involved in the prognosis of SCLC patients. Rs1051685 was related to the prognosis in non-metastasis patients. Conclusion: The *ERCC5* rs873601 (G>A) was a valuable biomarker for predicting the prognosis in lung cancer patients treated with platinum-based chemotherapy.

## 1. Introduction

Lung cancer is one of the leading cancers and the highest lethal malignancies in the world [1]. It consists of Small Cell Lung Cancer (SCLC) and Non-Small Cell Lung Cancer (NSCLC). NSCLC accounts for almost 80% of lung cancer cases, including adenocarcinoma, squamous cell cancer, and large cell lung cancer [2]. The treatment strategy for lung cancer consists of surgery, radiation oncology, chemotherapy, immunotherapy, and targeted therapy [3]. Despite the progress of immunotherapy and targeted therapy in the past years, platinum-based chemotherapy combined with immunotherapy is still the first-line treatment for lung cancer patients [4]. The 5-year survival is a crucial indicator of the treatment efficacy [5]. The occurrence of drug resistance and treatment toxicity creates substantial barriers to disease control, such as gastrointestinal toxicity and hematological toxicity, which result in poor 5-year survival [6]. Remarkably, the chemotherapy outcomes differ from individual, which means the genetic polymorphisms may play an important role in the efficacy of chemotherapy treatment [7]. Until now, plentiful genetic polymorphisms are associated with the outcomes of chemotherapy, such as Eukaryotic translation Initiation Factor 3 subunit A (*eIF3A*), Rac family small GTPase 1 (*RAC1*), WNT1 Inducible Signaling Pathway protein 1 (*WISP1*), and so on [8,9,10]. The specific mechanisms are still being discovered on the way.

As we all know, DNA damage and repair pathways are of great importance in health and disease [11]. DNA damage can be classified into two main categories based on its origin: endogenous and exogenous [12]. DNA damage and repair pathways can prevent DNA damage from causing mutations and cytotoxicity, but the unbalanced repair of DNA damage always leads to the development of tumors [13,14]. It has been reported that the genetic polymorphisms of DNA repair pathways can significantly affect the response to cisplatin treatment in lung cancer patients [15,16]. The DNA damage and repair pathway consists of mismatch repair (MMR), base excision repair (BER), nucleotide excision repair (NER), and double-strand break (DSB) repair systems [17].

The Excision Repair Cross-Complementation 5 (*ERCC5*), also called Xeroderma Pigmentosum Group (*XPG*), is a gene performing its function in nucleotide excision repair (NER), and it can also protect replication forks by promoting homologous recombination [18]. *ERCC5* contains 17 exons and spans 32 kb with a location of chromosome 13q33.1 [19]. It plays an essential role in the occurrences and clinical outcomes of lung cancer. The *ERCC5* rs4771436 and rs1047768 genotypes are associated with the risk of lung cancer [20]. The *ERCC5* His46His genomic polymorphisms can significantly affect the response to chemotherapy in advanced NSCLC patients [21].

The Xeroderma Pigmentosum group A (*XPA*) is another key member of NER, and it can catch the damage site of the DNA substrate by binding the NER core repair factors [22]. It is also reported to be connected with the development and efficacy of lung cancer. *XPA* rs1800975 polymorphisms have been reported to be associated with susceptibility in lung cancer patients [23]. The genomic variabilities of *XPA* rs2808668 are also considered to jointly contribute to lung cancer risk [24]. The mutation of *XPA* rs3176658 is significantly associated with progress-free survival in NSCLC patients [25].

The other genes of NER are also reported to play vital roles in lung cancer occurrences and clinical outcomes. The single nucleotide polymorphisms of *XRCC3* rs861539 are related to the prognosis of NSCLC patients [26]. The variables of *XRCC5* (rs1051685, rs6941) were associated with hematologic toxicity in lung cancer patients treated with platinum-based chemotherapy, which means it can predict platinum-based chemotherapy toxicity in lung cancer patients [27]. The expression of *ERCC1* may be a useful prognostic marker in lung adenocarcinoma, the lower expression had a longer overall survival [28]. Patients with the C/C genotype in rs3212986 of the *ERCC1* gene had longer median progress-free survival in NSCLC patients [29].

In this study, we selected 17 SNPs from ERCC5, PNKY, ERCC1, SIRT1, XPA, XRCC3, and XRCC5, such as rs873601, rs2444933, rs3740051, rs1869641, rs1051685, and so on. The rs873601 has been reported to be associated with cancer susceptibility [30]. The rs3740051 plays an important role in the development of pituitary adenoma [31]. The transporter gene polymorphisms of rs1869641 have been reported to show a significant relation to chemotherapy response [32]. The rs1051685 was reported to be associated with the response and survival in relapsed or refractory multiple myeloma patients [33]. Based on the previous study, we aim to find new biomarkers to predict the efficacy in lung cancer patients, which can make forward to more intensive guidance in the clinical diagnosis and treatment. 

## 2. Material and Methods

### 2.1. Research Objects and Treatment Procedures

All the subjects enrolled were selected with the following conditions: (1) patients who were diagnosed with lung cancer for the first time at Xiangya Hospital of Central South University or Hunan Province of Cancer Hospital (Changsha, Hunan, China) between August 2009 and January 2013; (2) patients did not receive surgery treatment before platinum-based chemotherapy. (3) all patients should receive at least 2 periods of platinum-based chemotherapy. The clinical characteristics of the enrolled subjects are listed in Table 1. All patients should sign the informed consent before they participate in this study. The investigation protocol was approved by the Ethics Committee of Xiangya School of Medicine, Central South University, with the registration number CTXY-110008-1. 

### 2.2. Data Collection 

The deadline for patients recruited was 15 July 2019. Their survival data were collected via telephone follow-up or residence registration. The endpoint criteria were progress-free survival (PFS) and overall survival (OS). The progress-free survival (PFS) was according to the diagnosis date of lung cancer and the date of the first local recurrence or metastases in the last follow-up. The overall survival (OS) time was calculated from the time between diagnosis of lung cancer and the date of the last follow-up or death. Patients without progression were defined as censors when analyzed. As researchers, the polymorphisms of the patients were unknown before the sequencing analysis. 

### 2.3. SNP Selecting, DNA Extraction and Genotyping

There were 17 common SNPs of *DNA* damage and repair genes selected in our study (Table 2). The candidate SNPs were located from 5 kb upstream of the first exon to the downstream of the last exon, respectively. We used Haploview version 4.2 to choose the Haplotype tagging SNPs. They were chosen based on our previous research about lung cancer prognosis [9,34], and these genes were also associated with the outcome of multiple cancers [35,36]. And all the selected SNPs must satisfy the condition that the minor allele frequency (MAF) > 0.05 in the HapMap CHB population. The DNA we used for genotyping was separated from a 5ml external blood sample using FlexiGene DNA Kit (Qiagen, Hilden, Germany). All the samples were stored at 4 °C before use. Genotyping was conducted by Sequenom’s MassARRAY system (Sequenom, San Diego, CA, USA).

### 2.4. Statistical Analysis

We used Cox proportional hazard models to analyze the differences in the variables, such as histology, age, clinical stage, smoking status, gender, and metastasis between the PFS and OS. We used the forward stepwise method of Cox proportional hazard models to find the covariates. Variables that were significantly associated with OS or PFS were considered as the covariates in the specific subgroup. And then, we fit the covariates into a multivariate logistic regression model to adjust the covariates via the command of -cover in PLINK. The *p*-value was 2-sided, and *p* < 0.05 will be considered statistically significant. All association analyses were conducted using three models, including additive, dominant, and recessive. The additive model is for the additive effects of SNPs. It means if D is a minor allele and d is the major allele, the additive model means DD vs. Dd vs. dd. Dominant and recessive models are tests for the minor allele with two of the classes pooled. The dominant model means (DD, Dd) vs. dd, and the recessive model means DD vs. (Dd, dd). The aforementioned statistical analyses were performed using PLINK (ver 1.07, https://zzz.bwh.harvard.edu/plink/, accessed on 10 October 2009) and SPSS 18.0 (SPSS Inc., Chicago, IL, USA).

## 3. Results

### 3.1. Distribution of Characteristics in Lung Cancer Patients and Prognosis Analysis

The demographic characteristics and prognosis consequences for the 345 lung cancer patients are provided in Table 3. The majority of these patients were NSCLC (67.5%), compared with SCLC (28.7%). The median age was 56 years old. Most of them were diagnosed at an advanced stage, III/IV/ED (86.4%), in contrast to I/II/LD (11.9%). More than half of the patients were smokers (64.6%), with a non-smoking proportion of 35.4%. Most patients were male (82.6%) versus female (17.4%). The patients with metastasis were 43.2%, and those without metastasis were 17.7%. The median survival time of overall survival (MST-OS) is 4.42 years, and the median survival time of progression-free survival (MST-PFS) is 3.16 years. The other statistics of the clinical outcomes in the above subgroups were also summarized inTable 3.

### 3.2. Association between Polymorphisms and Prognosis in the Lung Cancer Patients

**Figure 1:**
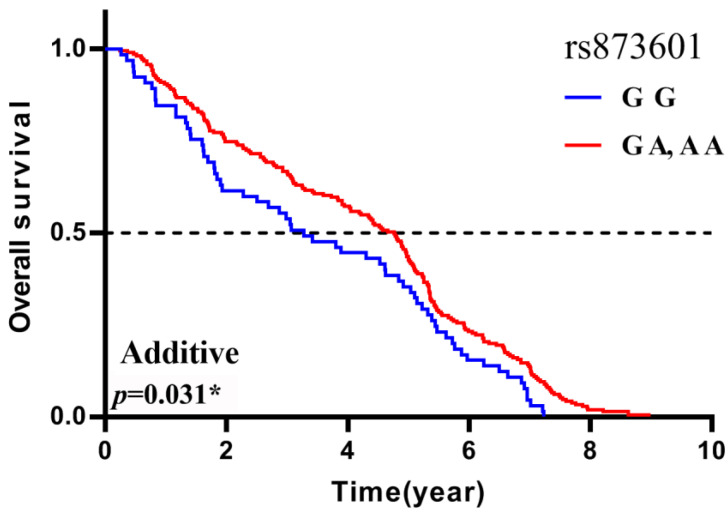
The *ERCC5* rs873601 polymorphisms are significantly associated with the prognosis in lung cancer patients treated with platinum-based chemotherapy, and the A variant allele of *ERCC5* rs873601 are protective allele. Patients who carry the AA or GA genotypes have a longer MST-OS than GG (*p* = 0.031 *). The dashed line, the overall survival rate is 0.5 or 50%.

As we analyzed, the genomic polymorphisms *ERCC5* rs873601 (G>A) were significantly associated with the overall survival (OS) of lung cancer patients in the recessive model (*p* = 0.031). This means that patients who carry the *ERCC5* rs873601 GG genotype had a shorter MST-OS than the patients who have the *ERCC5* rs873601 GA or AA genotypes (MST-OS: 3.28, 4.88, 4.02 years, respectively) (Table 4). The “BETA” in Table 4 and Table 5 means the estimated value in the logistic regression. In conclusion, patients who carry the allele A of *ERCC5* rs873601 are the protective allele in the prognosis of lung cancer treated with platinum-based chemotherapy (Figure 1).

### 3.3. Stratification Analyses of Association between Polymorphisms and Prognosis in Lung Cancer Patients

**Figure 2:**
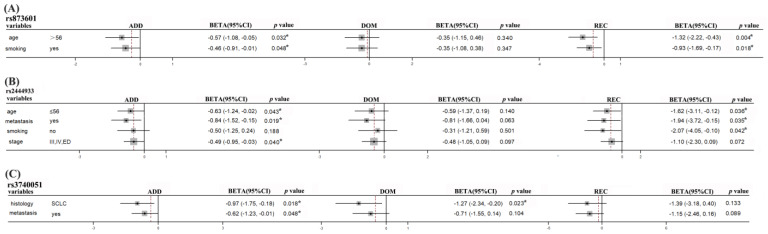
The polymorphisms of *ERCC5* rs873601, *PNKY* rs2444933, and *STIR1* rs1051685 were related to the overall survival (OS) significantly. (**A**) The *ERCC5* rs873601 polymorphisms were significantly associated with the overall survival (OS) in age > 56 and smoking patients. (**B**) The variants of *PNKY* rs2444933 were related to the prognosis significantly in age ≤ 56, metastasis, non-smoker, and clinical in III/IV/ED patients. (**C**) The polymorphisms of *STIR1* rs3740051 were significantly associated with the overall survival in SCLC and metastasis patients. Dashed line, merged BETA value. *, *p* < 0.05.

To further elucidate the association between these SNPs and the prognosis in lung cancer patients, we also performed subgroup analysis based on age, gender, smoking status, histology, clinical stage, and metastasis. As shown in Table 5, the *ERCC5* rs873601 was related to overall survival in additive and recessive model in age > 56 years lung cancer patients (Additive model: *p* = 0.032; Recessive model: *p* = 0.004) and smoker patients in additive and recessive model (Additive model: *p* = 0.048; Recessive model: *p* = 0.018). The *PNKY* rs2444933 was significantly associated with overall survival in age ≤ 56 (Additive model: *p* = 0.043; Recessive model: *p* = 0.036), metastasis (Additive model: *p* = 0.019; Recessive model: *p* = 0.035), non-smoking (Recessive model: *p* = 0.042) and III/IV/ED (Additive model: *p* = 0.040) patients. Furthermore, the *STIR1* rs3740051 polymorphisms in SCLC (Additive model: *p* = 0.018; Dominant model: *p* = 0.023) and metastasis (Additive model: *p* = 0.048) patients were significantly associated with overall survival (Figure 2).

**Figure 3:**
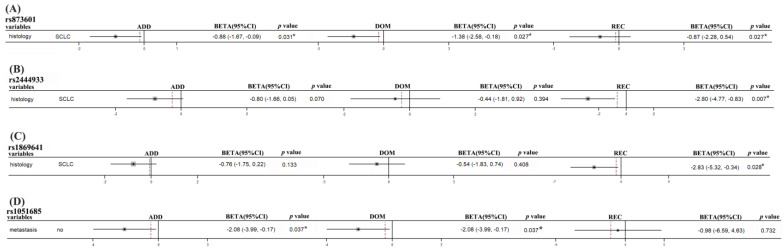
The polymorphisms of *ERCC5* rs873601, *PNKY* rs2444933, rs1849641, and *XRCC5* rs1051685 were significant with the progress-free survival (PFS) significantly. (**A**–**C**) The *ERCC5* rs873601, *PNKY* rs2444933, and rs1869641 polymorphisms were significantly associated with progression-free survival (PFS) in SCLC patients. (**D**) The variants of *XRCC5* rs1051685 were related to the prognosis significantly in non-metastasis patients. Dashed line, merged BETA value. *, *p* < 0.05.

We also analyzed the association between the genomic polymorphisms with progress-free survival (PFS) in subgroups. The polymorphisms of *ERCC5* rs873601 (Additive model: *p* = 0.031; Dominant model: *p* = 0.027), *PNKY* rs2444933 (Recessive model: *p* = 0.007), and *PNKY* rs1869641 (Recessive model: *p* = 0.028) were significantly associated with the PFS in SCLC patients. The *XRCC5* rs1051685 were significantly associated with the PFS in non-metastasis patients in additive and dominant models (Additive model: *p* = 0.037; Dominant model: *p* = 0.037) (Figure 3).

**Figure 4:**
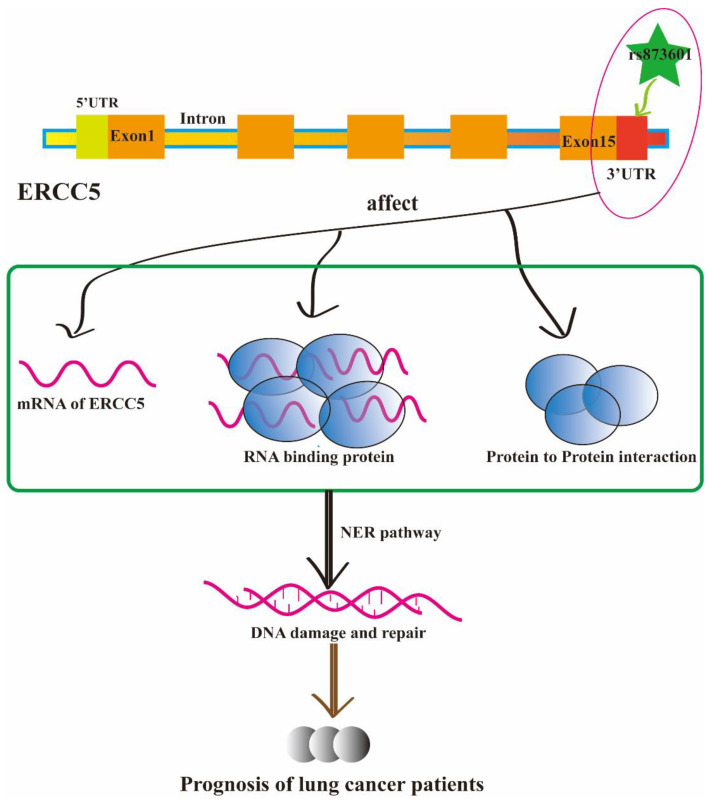
The polymorphisms of ERCC5 rs873601 can affect the prognosis of lung cancer patients. Rs873601 is a mutation located in the 3′UTR of ERCC5, it can affect the mRNA of ERCC5 to translation protein. It can also regulate the RNA-binding protein and the protein-to-protein interaction of ERCC5. These may have an influence on the DNA damage and repair in the NER pathway, which is a vital regulator in the prognosis of lung cancer patients treated with platinum-based chemotherapy.

As we showed in Figure 4, the main finding of our study was that the polymorphisms of *ERCC5* rs873601 play an important role in the prognosis of lung cancer patients treated with platinum-based chemotherapy. The mechanism of which is also valuable will be discussed. The rs873601 is a 3_prime_UTR_variant in ERCC5, and it may affect the translation of mRNA of ERCC5, the RNA-binding protein (RBP), and the protein-to-protein interaction. As we all know, the function of 3′UTR to regulate the mRNA stability or translation [37] and interactions between the mRNA and possible binding proteins have been widely reported [38]. It has also been reported that 3’UTR can regulate protein characteristics by mediating 3’UTR-dependent protein–protein interactions (PPI) [39]. 3’UTR can regulate the formation of protein complexes and determine the function of proteins, indicating that the genetic information encoded by the 3’ UTR can be transmitted to proteins [40]. Single-nucleotide polymorphisms (SNPs) in the 3’-terminal untranslated region (3’-UTR) targeted by microRNAs (miRNAs) can alter the gene function, which can impact the function of nucleotide excision repair (NER) pathway [41]. In other words, rs873601 is a 3’UTR variant in ERCC5, and it may alter the gene function of ERCC5. It can regulate the NER pathway of DNA damage and repair, which has been reported to be significantly associated with platinum-based chemotherapy resistance. ERCC5 rs873601 may be a potential therapeutic target in the treatment of lung cancer patients, which is valuable and will be further investigated.

## 4. Discussion

As a crucial member of the DNA damage and repair system, there are plenty of reports about *ERCC5* and lung cancer. The *ERCC5* rs17655GG was associated with an increased risk of lung cancer, and it may be associated with lung cancer susceptibility in the Chinese population [42]. In the other investigation, *ERCC5* rs4771436 and rs1047768 genotypes were reported to be associated with an increased risk of lung cancer patients [20]. *ERCC5* rs751402 polymorphisms were significantly related to the risk in NSCLC patients in North Indians [43]. In other populations of the coal-mining region, the genomic variants of *ERCC5* rs17655 were associated with lung cancer risk significantly [44]. There were also investigations about *ERCC5* and lung cancer prognosis, *ERCC5* (rs2094258 and rs2296147) was reported to be related to progression-free survival (PFS) in NSCLC patients treated with platinum-based chemotherapy [45]. It has been also found that the *ERCC5* rs751402 genotype was associated with the treatment response in patients with advanced non-small-cell lung cancer treated with platinum-based chemotherapy [46]. The SNPs of *ERCC5* in Nucleotide Excision Repair (NER) pathway genes were correlated with toxicity treated with double chemotherapy in advanced NSCLC patients [47]. These all mean that the polymorphisms in DNA repair genes are significantly related to the risk of lung cancer and play an important role in the occurrence of lung cancer. The novelty of our results is that we noticed the importance of polymorphisms in 3′UTR in *ERCC5* rather than the exon mutations in other studies. We found that the *ERCC5* rs873601 plays a critical role in the resistance of platinum-based chemotherapy in lung cancer patients, which can perfect the investigation of ERCC5 gene mutations in lung cancer. And it may provide new insights into the treatment of lung cancer patients.

As we all know, Human Epidermal growth factor Receptor 2 (*HER2/ERBB2*) and Epidermal Growth Factor Receptor (*EGFR*) are two crucial biomarkers in the prognosis of lung cancer [48,49]. These biomarkers are often used for the screening, detection, diagnosis, prognosis, prediction, and monitoring of cancer development [50]. It has been reported that the adverse drug reaction (ADR) in HER2 (+) patients with Grade 3 or 4 was significantly higher than that in the control group in NSCLC patients [51]. EGFR tyrosine kinase inhibitors (TKIs) are an important treatment regimen for lung cancer patients. However, up to 50% of patients treated with first- and second-generation TKIs develop an EGFR exon 20 T790M mutation at the time of progression, which may lead to a treatment failure in these patients [52]. Finding new biomarkers for lung cancer patients is of great importance. ERCC5 as an important component in the repair pathway of platinum-induced damage, plays an important role in the prognosis of lung cancer patients [53]. The polymorphisms of ERCC5 have been reported to be associated with the risk of NSCLC [54]. ERCC5 may become a potential therapeutic target for the treatment of lung cancer patients, as important as HER2 and EGFR.

We also found that *PNKY* rs2444933 and rs1869641 were associated with the prognosis in lung cancer patients through the stratified analysis. Most reports of *PNKY* were about its function in the brain, there are several investigations into its role in cancer. It has been found that *PNKY* can inhibit the binding of miR124 to Polypyrimidine Tract-Binding Protein 1 (*PTBP1*) and maintain the homeostasis of choroidal vascular function [55]. *PNKY* may control the resistance of platinum-based chemotherapy via the regulation of choroidal vascular function. *STIR1* rs3740051 and *XRCC5* rs1051685 polymorphisms were associated with the prognosis significantly in lung cancer patients treated with platinum-based chemotherapy. *STIR1* was reported to be related to immune evasion, which may be essential to maintain their stability [56]. *STIR1* may play an important role in lung cancer survival via its regulation of immune evasion. *XRCC5* was overexpressed in lung adenocarcinoma, it may be a risk factor, and it can also predict a poor prognosis in lung adenocarcinoma patients [57]. The other investigation also found that *XRCC5* was an independent risk factor affecting the prognosis of lung adenocarcinoma patients [58]. It was also reported that the transcriptional overexpression of *XRCC5* showed a significant correlation with a shorter patient outcome in advanced lung cancer patients [59].

Our study investigated the association between the polymorphisms of DNA repair genes, *PNKY* and *STIR1*, with the prognosis in Chinese lung cancer patients treated with platinum-based chemotherapy. We also stratified these polymorphisms in age, gender, smoking, histology, clinical stage, and metastasis. However, there were several limitations in our study. First, the sample size of our study was not large enough, as we only enrolled 345 patients in our project. As we performed multiple testing corrections, there were no significant SNPs remaining. Second, the biological function mechanisms of these SNPs need further study in vitro. Finally, the validation of our results needs replication studies with other independent subjects. 

In conclusion, the variants of *ERCC5* rs873601 were significantly associated with the prognosis in lung cancer patients treated with platinum-based chemotherapy. Patients carrying the *ERCC5* rs873601 A allele may have a longer overall survival (OS) than the G allele. The genotypes of *ERCC5* rs873601 may be an attractive biomarker used to predict the prognosis of lung cancer patients treated with platinum-based chemotherapy.

## Figures and Tables

**Table 1:** Clinical characteristics of lung cancer patients.

Patient Characteristics	N (%)
Total no. of patients	345
Histology	
Adenocarcinoma	112 (32.5)
Squamous cell	111 (32.2)
Small cell	99 (28.7)
Age	
≤56	172 (49.9)
>56	173 (50.1)
Clinical stage	
I/II/LD	41 (11.9)
III/IV/ED	298 (86.4)
Smoking status	
Non-smoker	122 (35.4)
Smoker	223 (64.6)
Gender	
Male	285 (82.6)
Female	60 (17.4)
Metastasis	
No	61 (17.7)
Yes	149 (34.2)
Chemotherapy regimens	
Platinum/gemcitabine	109 (31.6)
Platinum/paclitaxel	59 (17.1)
Platinum/navelbine	8 (2.3)
Platinum/etoposide	77 (22.3)
Platinum/irinotecan	8 (2.3)

**Table 2:** The 17 polymorphisms examined in this study.

Gene	Locus	dbSNP	Call Rate (%)	Polymorphism	MAF	Genotype	N (%)
*ERCC1*	19q13.32	rs12984195	97.97	T>C	3.70	TT	325 (94.2)
						TC	13 (3.8)
						CC	0
		rs117128015	100.00	C>T	8.00	CC	315 (91.3)
						CT	30 (8.7)
						TT	0
*ERCC5*	13q33.1	rs873601	99.13	G>A	49.11	GG	84 (24.4)
						GA	165 (47.8)
						AA	93 (27.0)
*PNKY*	6q16.1	rs1869641	98.84	G>A	22.22	GG	247 (71.6)
						GA	82 (23.8)
						AA	12 (3.5)
		rs1883306	93.91	T>G	38.53	TT	146 (42.3)
						TG	138 (40.0)
						GG	40 (11.6)
		rs2444933	99.13	A>G	31.62	AA	194 (56.2)
						AG	126 (36.5)
						GG	22 (6.4)
*SIRT1*	10q21.3	rs3758391	97.39	T>C	24.46	TT	248 (71.9)
						TC	74 (21.5)
						CC	14 (4.1)
		rs3740051	96.52	A>G	33.41	AA	182 (52.8)
						AG	119 (34.5)
						GG	32 (9.3)
		rs4746720	99.13	T>C	46.67	TT	118 (34.2)
						TC	138 (40.0)
						CC	86 (24.9)
		rs12778366	95.65	T>C	22.09	TT	239 (69.3)
						TC	82 (23.8)
						CC	9 (2.6)
*XPA*	9q22.33	rs3176751	98.84	G>C	20.61	GG	2 (0.6)
						GC	86 (24.9)
						CC	253 (73.3)
		rs3176752	98.84	C>A	21.44	CC	249 (72.2)
						CA	88 (25.5)
						AA	4 (1.2)
*XRCC3*	14q32.33	rs3212117	98.84	C>A	5.28	CC	322 (93.3)
						CA	19 (5.5)
						AA	0
		rs3212121	98.55	A>G	4.24	AA	325 (94.2)
						AG	14 (4.1)
						GG	1 (0.3)
*XRCC5*	2q35	rs1051677	98.84	T>C	22.43	TT	245 (71.0)
						TC	87 (25.2)
						CC	9 (2.6)
		rs2440	98.55	C>T	34.03	CC	24 (7.0)
						CT	139 (40.3)
						TT	177 (51.3)
		rs1051685	99.13	A>G	12.14	AA	295 (85.5)
						AG	45 (13.0)
						GG	2 (0.6)

**Table 3:** Distribution of characteristics in lung cancer patients and prognosis analysis.

Variables	Patients N (%)	Death N (%)	MST-OS (Year)	MST-PFS (Year)
Lung cancer	345	279	4.42	3.16
NSCLC	233 (67.5)	188 (67.4)	4.56	3.25
SCLC	99 (28.7)	80 (28.7)	4.17	3.10
Age				
≤56	172 (49.9)	142 (50.9)	4.48	2.95
>56	173 (50.1)	137 (49.1)	4.36	3.80
Clinical stage				
I/II/LD	41 (11.9)	31 (11.1)	3.14	4.05
III/IV/ED	298 (86.4)	243 (87.1)	4.55	3.21
Smoking status				
Non-smoker	122 (35.4)	97 (34.8)	4.77	4.60
Smoker	223 (64.6)	182 (65.2)	4.26	2.61
Gender				
male	285 (82.6)	229 (82.1)	4.39	2.93
female	60 (17.4)	50 (17.9)	4.57	4.47
Metastasis				
No	61 (17.7)	51 (18.3)	3.84	2.28
Yes	149 (43.2)	121 (43.4)	4.53	3.94

MST, median survival time; OS, overall survival; PFS, progression-free survival; NSCLC, Non-Small Lung Cancer; SCLC, Small Cell Lung Cancer; LD, limitation period; ED, extensive period.

**Table 4:** Association of the ERCC5 rs873601 polymorphisms and OS in lung cancer patients.

Gene	Polymorphisms	Genotype	MST (Year)	Additive		Dominant		Recessive	
				BETA (95%CI)	*p* Value	BETA (95%CI)	*p* Value	BETA (95%CI)	*p* Value
*ERCC5*	rs873601	G G	3.28	−0.37 (−0.75, 0.01)	0.061	−0.30 (−0.91, 0.32)	0.348	−0.70 (−1.34, −0.07)	0.031 *
		G A	4.88						
		A A	4.02						

*, *p* < 0.05.

**Table 5:** Stratification analyses of association between polymorphisms and OS or PFS in lung cancer patients.

OS/PFS	Gene	Polymorphisms	Subgroup	Additive		Dominant		Recessive	
				BETA (95%CI)	*p* Value	BETA (95%CI)	*p* Value	BETA (95%CI)	*p* Value
OS	*ERCC5*	rs873601	age > 56	−0.57 (−1.08, −0.05)	0.032 *	−0.35 (−1.15, 0.46)	0.399	−1.32 (−2.22, −0.43)	0.004 *
			smokers	−0.46 (−0.91, −0.01)	0.048 *	−0.35 (−1.08, 0.38)	0.347	−0.93 (−1.69, −0.17)	0.018 *
	*PNKY*	rs2444933	age ≤ 56	−0.63 (−1.24, −0.02)	0.043 *	−0.59 (−1.37, 0.19)	0.140	−1.62 (−3.11, −0.12)	0.036 *
			metastasis	−0.84 (−1.53, −0.15)	0.019 *	−0.81 (−1.66, 0.04)	0.063	−1.94 (−3.72, −0.15)	0.035 *
			non-smoker	−0.51 (−1.25, 0.24)	0.188	−0.31 (−1.21, 0.59)	0.501	−2.07 (−4.05, −0.10)	0.042 *
			III/IV/ED	−0.49 (−0.95, −0.03)	0.040 *	−0.48 (−1.05, 0.09)	0.097	−1.10 (−2.30, 0.09)	0.072
	*STIR1*	rs3740051	SCLC	−0.97 (−1.75, −0.18)	0.018 *	−1.27 (−2.34, −0.20)	0.023 *	−1.39 (−3.18, 0.40)	0.133
			metastasis	−0.62 (−1.23, −0.01)	0.048 *	−0.71 (−1.55, 0.14)	0.104	−1.15 (−2.46, 0.16)	0.089
PFS	*ERCC5*	rs873601	SCLC	−0.88 (−1.67, −0.09)	0.031 *	−1.38 (−2.58, −0.18)	0.027 *	−0.87 (−2.28, 0.54)	0.230
	*PNKY*	rs2444933	SCLC	−0.80 (−1.66, 0.05)	0.070	−0.49 (−1.60, 0.63)	0.394	−2.80 (−4.77, −0.83)	0.007 *
		rs1869641	SCLC	−0.76 (−1.75, 0.22)	0.133	−0.54 (−1.83, 0.74)	0.408	−2.83 (−5.32, −0.34)	0.028 *
	*XRCC5*	rs1051685	Non-metastasis	−2.08 (−3.99, −0.17)	0.037 *	−2.08 (−3.99, −0.17)	0.037 *		

*, *p* < 0.05.

## Data Availability

The original data is unavailable due to privacy or ethical restrictions, if you are interest in the original data, you can contact me directly.

## References

[1] Siegel R.L., Miller K.D., Fuchs H.E., Jemal A. (2022). Cancer statistics, 2022. CA Cancer J. Clin..

[2] Roy-Chowdhuri S. (2021). Molecular Pathology of Lung Cancer. Surg. Pathol. Clin..

[3] Vrana D. (2021). Advances in the therapy of small cell lung cancer. Klin. Onkol..

[4] Miller M., Hanna N. (2021). Advances in systemic therapy for non-small cell lung cancer. BMJ.

[5] Schulz C. (2021). Advances in Lung Cancer Treatment. Dtsch. Med. Wochenschr..

[6] Park S., Lee H., Lee B., Lee S.H., Sun J.M., Park W.Y., Ahn J.S., Ahn M.J., Park K. (2019). DNA Damage Response and Repair Pathway Alteration and Its Association with Tumor Mutation Burden and Platinum-Based Chemotherapy in SCLC. J. Thorac. Oncol..

[7] Ge F., Huo Z., Li C., Wang R., Wang R., Liu Y., Chen J., Lu Y., Wen Y., Jiang Y. (2021). Lung cancer risk in patients with multiple sclerosis: A Mendelian randomization analysis. Mult. Scler. Relat. Disord..

[8] Zou T., Yin J., Zheng W., Xiao L., Tan L., Chen J., Wang Y., Li X., Qian C., Cui J. (2016). Rho GTPases: RAC1 polymorphisms affected platinum-based chemotherapy toxicity in lung cancer patients. Cancer Chemother. Pharmacol..

[9] Yin J.Y., Zhang J.T., Zhang W., Zhou H.H., Liu Z.Q. (2018). eIF3a: A new anticancer drug target in the eIF family. Cancer Lett..

[10] He J., Wang Z., Wang Y., Zou T., Li X.P., Cao L., Chen J. (2021). The Effects of WISP1 Polymorphisms on the Prognosis of Lung Cancer Patients with Platinum-Based Chemotherapy. Pharmacogenom. Pers. Med..

[11] Schumacher B., Pothof J., Vijg J., Hoeijmakers J.H.J. (2021). The central role of DNA damage in the ageing process. Nature.

[12] Ashour M.E., Mosammaparast N. (2021). Mechanisms of damage tolerance and repair during DNA replication. Nucleic Acids Res..

[13] Kay J., Thadhani E., Samson L., Engelward B. (2019). Inflammation-induced DNA damage, mutations and cancer. DNA Repair.

[14] Paull T.T. (2021). DNA damage and regulation of protein homeostasis. DNA Repair.

[15] Dai C.H., Li J., Chen P., Jiang H.G., Wu M., Chen Y.C. (2015). RNA interferences targeting the Fanconi anemia/BRCA pathway upstream genes reverse cisplatin resistance in drug-resistant lung cancer cells. J. Biomed. Sci..

[16] Pilger D., Seymour L.W., Jackson S.P. (2021). Interfaces between cellular responses to DNA damage and cancer immunotherapy. Genes Dev..

[17] Gorbunova V., Seluanov A., Mao Z., Hine C. (2007). Changes in DNA repair during aging. Nucleic Acids Res..

[18] Muniesa-Vargas A., Theil A.F., Ribeiro-Silva C., Vermeulen W., Lans H. (2022). XPG: A multitasking genome caretaker. Cell. Mol. Life Sci..

[19] Kiyohara C., Yoshimasu K. (2007). Genetic polymorphisms in the nucleotide excision repair pathway and lung cancer risk: A meta-analysis. Int. J. Med. Sci..

[20] Lan X., Li Y., Wu Y., Li X., Xu L. (2022). The Association of ERCC1 and ERCC5 Polymorphisms with Lung Cancer Risk in Han Chinese. J. Cancer.

[21] Liu D., Wu J., Shi G.Y., Zhou H.F., Yu Y. (2014). Role of XRCC1 and ERCC5 polymorphisms on clinical outcomes in advanced non-small cell lung cancer. Genet. Mol. Res..

[22] Pradhan S., Sarma H., Mattaparthi V.S.K. (2019). Investigation of the probable homo-dimer model of the Xeroderma pigmentosum complementation group A (XPA) protein to represent the DNA-binding core. J. Biomol. Struct. Dyn..

[23] Yuan M., Yu C., Yu K. (2020). Association of human XPA rs1800975 polymorphism and cancer susceptibility: An integrative analysis of 71 case-control studies. Cancer Cell Int..

[24] Mei C., Hou M., Guo S., Hua F., Zheng D., Xu F., Jiang Y., Li L., Qiao Y., Fan Y. (2014). Polymorphisms in DNA repair genes of XRCC1, XPA, XPC, XPD and associations with lung cancer risk in Chinese people. Thorac. Cancer.

[25] Song X., Wang S., Hong X., Li X., Zhao X., Huai C., Chen H., Gao Z., Qian J., Wang J. (2017). Single nucleotide polymorphisms of nucleotide excision repair pathway are significantly associated with outcomes of platinum-based chemotherapy in lung cancer. Sci. Rep..

[26] Su Y., Zhang H., Xu F., Kong J., Yu H., Qian B. (2015). DNA Repair Gene Polymorphisms in Relation to Non-Small Cell Lung Cancer Survival. Cell. Physiol. Biochem..

[27] Chen J., Wu L., Wang Y., Yin J., Li X., Wang Z., Li H., Zou T., Qian C., Li C. (2016). Effect of transporter and DNA repair gene polymorphisms to lung cancer chemotherapy toxicity. Tumor Biol..

[28] Piljic Burazer M., Mladinov S., Matana A., Kuret S., Bezic J., Glavina Durdov M. (2019). Low ERCC1 expression is a good predictive marker in lung adenocarcinoma patients receiving chemotherapy based on platinum in all TNM stages—A single-center study. Diagn. Pathol..

[29] Grenda A., Blach J., Szczyrek M., Krawczyk P., Nicos M., Kuznar Kaminska B., Jakimiec M., Balicka G., Chmielewska I., Batura-Gabryel H. (2020). Promoter polymorphisms of TOP2A and ERCC1 genes as predictive factors for chemotherapy in non-small cell lung cancer patients. Cancer Med..

[30] Huang J., Liu X., Tang L.L., Long J.T., Zhu J., Hua R.X., Li J. (2017). XPG gene polymorphisms and cancer susceptibility: Evidence from 47 studies. Oncotarget.

[31] Liutkeviciene R., Vilkeviciute A., Morkunaite G., Glebauskiene B., Kriauciuniene L. (2019). SIRT1 (rs3740051) role in pituitary adenoma development. BMC Med. Genet..

[32] Wang Y., Yin J.Y., Li X.P., Chen J., Qian C.Y., Zheng Y., Fu Y.L., Chen Z.Y., Zhou H.H., Liu Z.Q. (2014). The association of transporter genes polymorphisms and lung cancer chemotherapy response. PLoS ONE.

[33] Cibeira M.T., de Larrea C.F., Navarro A., Diaz T., Fuster D., Tovar N., Rosinol L., Monzo M., Blade J. (2011). Impact on response and survival of DNA repair single nucleotide polymorphisms in relapsed or refractory multiple myeloma patients treated with thalidomide. Leuk. Res..

[34] Zou T., Liu J.Y., She L., Yin J.Y., Li X., Li X.P., Zhou H.H., Chen J., Liu Z.Q. (2020). The Association Between Heat-Shock Protein Polymorphisms and Prognosis in Lung Cancer Patients Treated With Platinum-Based Chemotherapy. Front. Pharmacol..

[35] Qin X.P., Zhou Y., Chen Y., Li N.N., Wu X.T. (2014). XRCC3 Thr241Met polymorphism and gastric cancer susceptibility: A meta-analysis. Clin. Res. Hepatol. Gastroenterol..

[36] Du P., Li G., Wu L., Huang M. (2022). Perspectives of ERCC1 in early-stage and advanced cervical cancer: From experiments to clinical applications. Front. Immunol..

[37] Rasekhian M., Roohvand F., Habtemariam S., Marzbany M., Kazemimanesh M. (2021). The Role of 3’UTR of RNA Viruses on mRNA Stability and Translation Enhancement. Mini-Rev. Med. Chem..

[38] Xu E., Zhang J., Zhang M., Jiang Y., Cho S.J., Chen X. (2014). RNA-binding protein RBM24 regulates p63 expression via mRNA stability. Mol. Cancer Res..

[39] Mayr C. (2019). What Are 3′ UTRs Doing?. Cold Spring Harb. Perspect. Biol..

[40] Berkovits B.D., Mayr C. (2015). Alternative 3′ UTRs act as scaffolds to regulate membrane protein localization. Nature.

[41] Gu S., Rong H., Zhang G., Kang L., Yang M., Guan H. (2016). Functional SNP in 3′-UTR MicroRNA-Binding Site of ZNF350 Confers Risk for Age-Related Cataract. Hum. Mutat..

[42] Li X., Zhang J., Su C., Zhao X., Tang L., Zhou C. (2012). The association between polymorphisms in the DNA nucleotide excision repair genes and RRM1 gene and lung cancer risk. Thorac. Cancer.

[43] Bhat G.R., Sethi I., Bhat A., Verma S., Bakshi D., Sharma B., Nazir M., Dar K.A., Abrol D., Shah R. (2021). Genetic evaluation of the variants using MassARRAY in non-small cell lung cancer among North Indians. Sci. Rep..

[44] Minina V.I., Bakanova M.L., Soboleva O.A., Ryzhkova A.V., Titov R.A., Savchenko Y.A., Sinitsky M.Y., Voronina E.N., Titov V.A., Glushkov A.N. (2019). Polymorphisms in DNA repair genes in lung cancer patients living in a coal-mining region. Eur. J. Cancer Prev..

[45] Perez-Ramirez C., Canadas-Garre M., Molina M.A., Robles A.I., Faus-Dader M.J., Calleja-Hernandez M.A. (2017). Contribution of genetic factors to platinum-based chemotherapy sensitivity and prognosis of non-small cell lung cancer. Mutat. Res. Rev. Mutat. Res..

[46] He C., Duan Z., Li P., Xu Q., Yuan Y. (2013). Role of ERCC5 promoter polymorphisms in response to platinum-based chemotherapy in patients with advanced non-small-cell lung cancer. Anticancer Drugs.

[47] Zhang L., Gao G., Li X., Ren S., Li A., Xu J., Zhang J., Zhou C. (2012). Association between single nucleotide polymorphisms (SNPs) and toxicity of advanced non-small-cell lung cancer patients treated with chemotherapy. PLoS ONE.

[48] Landi L., Cappuzzo F. (2013). HER2 and lung cancer. Expert Rev. Anticancer. Ther..

[49] Leonetti A., Sharma S., Minari R., Perego P., Giovannetti E., Tiseo M. (2019). Resistance mechanisms to osimertinib in EGFR-mutated non-small cell lung cancer. Br. J. Cancer.

[50] Sharma B., Kanwar S.S. (2018). Phosphatidylserine: A cancer cell targeting biomarker. Semin. Cancer Biol..

[51] Diao W.Y., Ding C.L., Yuan B.Y., Li Z., Sun N., Huang J.B. (2021). Clinical Characteristics and Prognosis of HER2 Gene Phenotype in Patients with Non-Small Cell Lung Cancer. Int. J. Gen. Med..

[52] Baraibar I., Mezquita L., Gil-Bazo I., Planchard D. (2020). Novel drugs targeting EGFR and HER2 exon 20 mutations in metastatic NSCLC. Crit. Rev. Oncol. Hematol..

[53] Abdalkhalek E.S., Wakeel L.M.E., Nagy A.A., Sabri N.A. (2022). Variants of ERCC5 and the outcome of platinum-based regimens in non-small cell lung cancer: A prospective cohort study. Med. Oncol..

[54] Zienolddiny S., Campa D., Lind H., Ryberg D., Skaug V., Stangeland L., Phillips D.H., Canzian F., Haugen A. (2006). Polymorphisms of DNA repair genes and risk of non-small cell lung cancer. Carcinogenesis.

[55] Shi L., Han X., Liu C., Li X., Lu S., Jiang Q., Yao J. (2022). Long Non-Coding RNA PNKY Modulates the Development of Choroidal Neovascularization. Front. Cell Dev. Biol..

[56] Liu T., Yang Q., Wei W., Wang K., Wang E. (2020). Toll/IL-1 receptor-containing proteins STIR-1, STIR-2 and STIR-3 synergistically assist Yersinia ruckeri SC09 immune escape. Fish Shellfish Immunol..

[57] Fan Y., Gao Z., Li X., Wei S., Yuan K. (2021). Gene expression and prognosis of x-ray repair cross-complementing family members in non-small cell lung cancer. Bioengineered.

[58] Zhang Q.X., Yang Y., Yang H., Guo Q., Guo J.L., Liu H.S., Zhang J., Li D. (2021). The roles of risk model based on the 3-XRCC genes in lung adenocarcinoma progression. Transl. Cancer Res..

[59] Saviozzi S., Ceppi P., Novello S., Ghio P., Lo Iacono M., Borasio P., Cambieri A., Volante M., Papotti M., Calogero R.A. (2009). Non-small cell lung cancer exhibits transcript overexpression of genes associated with homologous recombination and DNA replication pathways. Cancer Res..

